# Treatment Outcomes and Prognostic Factors of Patients With Primary Spinal Ewing Sarcoma/Peripheral Primitive Neuroectodermal Tumors

**DOI:** 10.3389/fonc.2019.00555

**Published:** 2019-06-25

**Authors:** Jun Chen, Mengxue Li, Yifeng Zheng, Lei Zheng, Fanfan Fan, Yu Wang

**Affiliations:** ^1^Department of Neurosurgery, Tongji Medical College, Tongji Hospital, Huazhong University of Science and Technology, Wuhan, China; ^2^Department of Ultrasonics, The 991th Hospital of the Joint Logistics Support Unit of the Chinese People's Liberation Army, Xiangyang, China; ^3^Department of Orthopedics, Tongji Medical College, Tongji Hospital, Huazhong University of Science and Technology, Wuhan, China

**Keywords:** Ewing sarcoma, primitive neuroectodermal tumors, survival, prognostic factor, spinal

## Abstract

**Purpose:** Primary spinal Ewing sarcoma (ES)/peripheral primitive neuroectodermal tumors (pPNETs) are extremely rare, and the current understanding of these tumors is poor. The authors aimed to illustrate the clinical characteristics of primary spinal ES/pPNETs and to discuss prognostic factors by survival analysis.

**Methods:** A total of 40 patients who were pathologically diagnosed with primary spinal ES/pPNETs between 2000 and 2018 were enrolled in this study. Progression-free survival (PFS) and overall survival (OS) were estimated by the Kaplan–Meier method to identify potential prognostic factors. Factors of *p* ≤ 0.1 in the Log-rank tests were subjected to multivariate analysis by Cox regression analysis.

**Results:** The mean follow-up period was 23.8 (range, 2–93) months, and 24 (60.0%) patients had local recurrence and 11 (27.5%) patients had distant metastasis. The 1-, 2-, and 5-year PFS rates were 57.7, 30.4, and 9.5%, respectively. The 1-, 2-, and 5-year OS rates were 74.8, 50.7, and 12.2%, respectively. The univariate analysis suggested that resection mode, postoperative Frankel score, adjuvant chemotherapy and adjuvant radiotherapy were potential prognostic factors for OS and PFS. However, after these factors were subjected to multivariate analyses, only adjuvant radiotherapy and resection mode remained as independent prognostic factors.

**Conclusions:** Total en bloc resection can significantly improve PFS for primary spinal ES/pPNETs and adjuvant radiotherapy was a favorable factor for PFS. Total en bloc resection and adjuvant radiotherapy considerably improve OS for patients with primary spinal ES/pPNETs.

## Introduction

Primary spinal Ewing sarcoma (ES)/peripheral primitive neuroectodermal tumors (pPNETs) are regarded as undifferentiated malignant small round cell tumors, which mostly occur in long bones, flat bones, ribs, and soft tissue. ES/pPNETs account for 6–8% of primary malignant bone tumors, and rarely affect intraspinal/vertebral deep mesenchymal/meningeal tissue (1–3). Due to a lack of clinic symptoms and specific biomarkers at the early stages of primary spinal ES/pPNETs, most patients are not diagnosed until advanced stages, which concomitantly worsens outcomes. Furthermore, because the tumor has an aggressive clinical course—with a high tendency for both local recurrence and distant metastasis—a timely and accurate preoperative diagnosis of primary spinal ES/pPNETs could provide useful information for surgical planning. Therefore, comprehensive studies on the clinical characteristics of primary spinal ES/pPNETs are warranted.

The rarity of the disease makes its purported surgical management and prognostic factors controversial. In addition, most related information about this disease comes from individual case reports or small case-series reports, which lack robust statistical outcomes. To illustrate the surgical management and prognostic factors of primary spinal ES/pPNETs, we retrospectively reviewed all of the cases surgically treated and pathologically confirmed as primary spinal ES/pPNETs at our institution between 2000 and 2018. Clinical, radiological, and pathological factors associated with longer progression-free survival (PFS) and overall survival (OS) were also analyzed.

## Materials and Methods

A total of 40 patients were surgically treated in Tongji Hospital (Tongji Medical College, Huazhong University of Science and Technology) between February 2000 and November 2018. All cases were analyzed by two experienced independent neuropathologist and were diagnosed according to the World Health Organization (WHO) classification of tumors. Clinical and spinal MRI follow-up data for patients with spinal ES/pPNETs were mainly obtained through outpatient review, supplemented by a telephone interview. Regular assessments were performed at 3, 6, and 12 months after initial surgery, every 6 months for the next 2 years, and then annually for life. The clinical data and surgical records for patients of primary spinal ES/pPNETs were retrospectively reviewed. Preoperative and postoperative neurologic statuses were classified according to the Frankel score (4). In the present study, all of the cases were divided into the following two subtypes: vertebral type and spinal canal type. The vertebral type was defined as any case in which the maximum diameter of the lesion was located in the vertebral body or accessory. The spinal canal type was defined as any case in which the maximum diameter of the lesion was located in the spinal canal.

Adjuvant treatment consisting of chemotherapy and/or radiotherapy was performed based on the patient's postoperative Karnofsky performance status (KPS) scores, age, preference, and tolerance. Patients with postoperative KPS scores ≥ 70 were recommended to undergo chemotherapy. Radiotherapy was performed in patients whose age was more than 3 years and who were unwilling to receive chemotherapy. In patients treated with chemotherapy, radiotherapy was performed based on the patient's age, preference, and tolerance. The vincristine, ifosfamide, doxorubicin, etoposide (VIDE) or vincristine, doxorubicin, cyclophosphamide (VAC) protocol was suggested for chemotherapy. We performed radiotherapy on the tumor resection site and the radiation dose ranged from 40 to 55 Gy.

The objective of this study was to illustrate the clinical, radiological, and pathological features of primary spinal ES/pPNETs and to discuss prognostic factors by survival analysis. PFS was defined as the time from the initial surgery to the time of the first event (i.e., tumor progression or death). The diagnosis of progression—including tumor recurrence, distant metastasis, and regrowth—was made on the basis of clinical presentations and imaging manifestations (e.g., enhanced magnetic resonance imaging or computed tomography scans). OS was defined as the time from the initial surgery to the date of death from any cause. The length of follow-up was recorded as the period from the date of the initial operation to death, or until November 2018 for surviving patients.

### Statistical Analysis

The univariate and multivariate analyses of various clinical, radiological, and pathological factors were performed to identify possible variables which could predict PFS and OS. The patient factors included age, gender, disease duration, preoperative Frankel score, and postoperative Frankel score. Tumor factors included subtype, involved segments, Ki67 index, bone destruction, and distant metastasis. The treatment factors were resection mode, postoperative radiotherapy, postoperative chemotherapy, and intraoperative blood loss. PFS and OS were evaluated by the Kaplan-Meier method to identify possible prognostic factors. Differences between survival curves were compared by using a log-rank test. Factors with *p* ≤ 0.1 in the log-rank tests were subjected to multivariate analysis by Cox regression analysis. We regarded *p* < 0.05 as statistically significant. Data were analyzed using SPSS version 20.0 package software (IBM Corp., Armonk, New York, USA).

## Results

### Patient Descriptions

The basic information of 40 patients is described in Tables 1, 2. The present study consisted of 24 (60%) males and 16 (40%) females with an average age of 21.9 (range, 1–45) years. The mean duration of the initial symptoms was 42 days (range 3–180 days). In our series, 28 (70%) patients presented with varied degrees of limb weakness, 20 (50%) patients presented with pain, and eight (20%) patients presented with incontinence.

**Table 1:** Radiological characteristics of 40 patients with primary spinal ES/pPNETs.

**Characteristic**	**No. of cases (%)**
**Location**	
Cervical only	5 (12.5)
Thoracic only	19 (47.5)
Lumbar only	6 (15.0)
Sacrum only	2 (5.0)
Cervical and thoracic	1 (2.5)
Thoracic and lumbar	4 (10.0)
Lumbar and sacrum	3 (7.5)
**Number of involved segments**	
Single	7 (17.5)
Multiple	33 (82.5)
**Subtype**	
Spinal canal type	32 (80.0)
Vertebral type	8 (20.0)
**Border of tumor**	
Well defined	25 (62.5)
Poorly defined	15 (37.5)
**T1 And T2 Signals**	
Hypointense T1 and isointense T2	8 (20.0)
Hypointense T1 and hyperintense T2	27 (67.5)
Isointense T1 and hyperintense T2	5 (12.5)
**Enhancement**	
Homogeneous	5 (12.5)
Heterogeneous	35 (87.5)
**Bone destruction**	
Yes	17 (42.5)
No	23 (57.5)

**Table 2:** Patient characteristics and univariate analysis of prognostic factors affecting progression-free survival and overall survival.

**Factors**	**Number**	**Progression-free survival**		**Overall survival**	
		**Median time (month)**	***p* value**	**Median time (month)**	***p* value**
**Age**					
<20/≥20 (year)	18/22	13 vs. 14	0.411	25 vs. 23	0.206
**Gender**					
Male/female	24/16	13 vs. 15	0.839	25 vs. 23	0.940
**Disease duration**					
<2/≥2 (month)	25/15	14 vs. 15	0.318	25 vs. 21	0.171
**Preoperative frankel score**					
A–C/D–E	26/14	13 vs. 15	0.487	23 vs. 27	0.436
**Subtype**					
Spinal canal type/vertebral type	32/8	15 vs. 8	0.329	25 vs. 18	0.481
**Number of involved segments**					
<3/≥3	12/28	13 vs. 15	0.572	25 vs. 25	0.931
**Resection mode**					
Total en bloc/total piecemeal/STR/PR	6/13/17/4	48 vs. 20 vs. 8 vs. 3	<0.001	55 vs. 28 vs. 18 vs. 7	<0.001
**KI-67 index**					
≤30/>30%	24/16	18 vs. 11	0.160	25 vs. 18	0.235
**Adjuvant radiotherapy**					
Yes/no	25/15	18 vs. 7	0.001	26 vs. 10	0.001
**Adjuvant chemotherapy**					
Yes/no	28/12	15 vs. 9	0.016	25 vs. 18	0.029
**Postoperative frankel score**					
A–C/D–E	13/27	11 vs. 15	0.019	18 vs. 25	0.013
**Intraoperative blood loss**					
<1,500/≥1,500 (mL)	19/21	15 vs. 14	0.972	25 vs. 23	0.991
**Bone destruction**					
Yes/no	17/23	9 vs. 15	0.386	18 vs. 26	0.285
**Distant metastasis**					
Yes/no	11/29	–	–	10 vs. 25	0.036

*STR, subtotal resection; PR, partial resection*.

**Figure 1:**
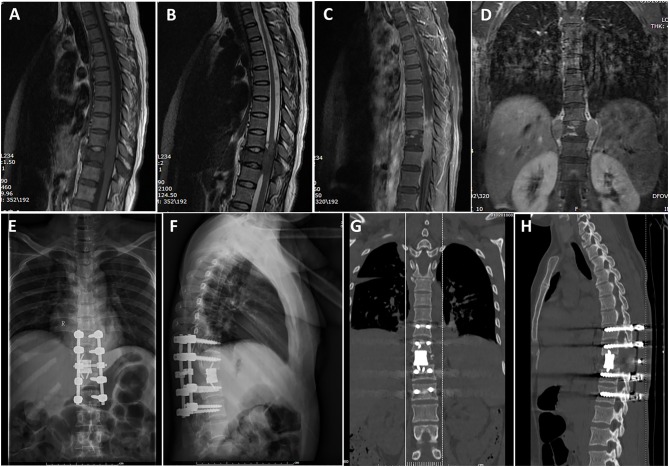
A case labeled as spinal canal type because the maximal diameter of the tumor was located in the spinal canal. Preoperative T1-weighted **(A)** and T2-weighted **(B)** images revealed a tumor at the T10–12 level. Contrast-enhanced sagittal **(C)** and coronary **(D)** images revealed that the tumor showed heterogeneous enhancement. Postoperative X-ray showed sound reconstruction by a 3D printed microporous titanium vertebral body and posterior screw-rod system. Anterior-posterior view **(E)**. Lateral view **(F)**. Postoperative computed tomographic scan of the thoracic spine 1 year after surgery showing excellent spinal fusion and the absence of tumor recurrence. Coronal section image **(G)**. Sagittal section image **(H)**.

**Figure 2:**
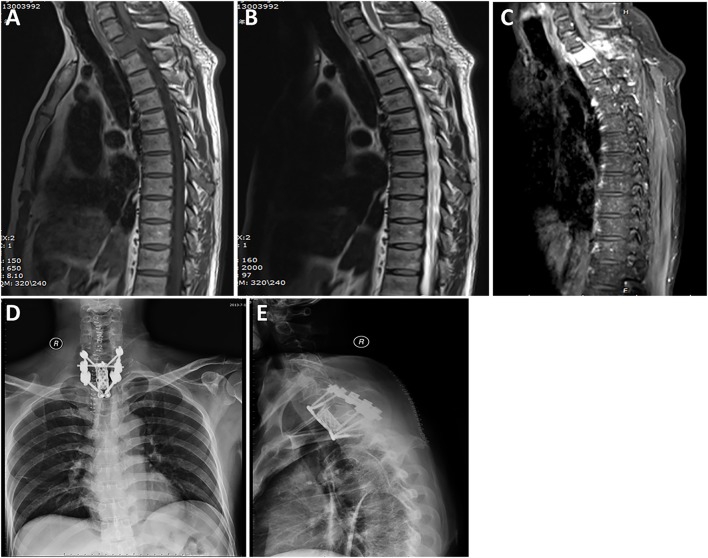
A case labeled as vertebral type because the maximal diameter of the tumor was located in the vertebral body and accessory. Preoperative T1-weighted **(A)** and T2-weighted **(B)** images revealed a tumor at the T1 level. Contrast-enhanced sagittal **(C)** image revealed that the tumor showed significant homogeneous enhancement. Postoperative radiograph of the thoracic spine after surgery showing that the reconstructed thoracic spine was well-maintained. Anterior-posterior view **(D)**. Lateral view **(E)**.

**Figure 3:**
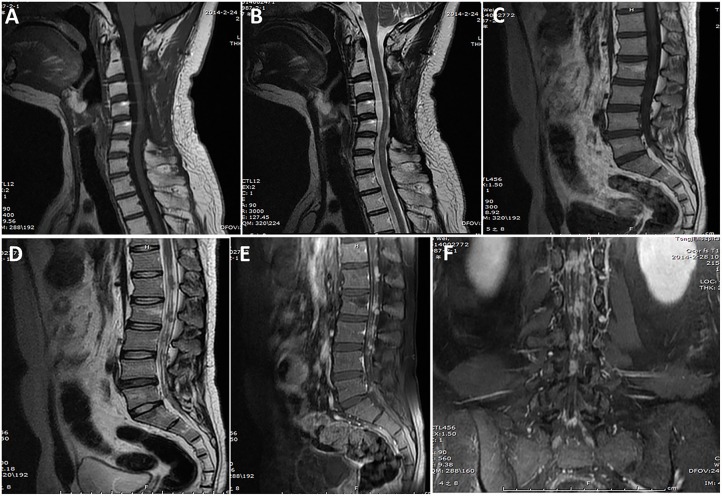
A case of primary intradural ES/pPNET at the C3–C5 level. Images obtained 14 months after the first surgery **(A,B)** showed no tumor local recurrence at the C3–5 level (lack of preoperative MRI examination findings), but they did show multiple metastases in the spinal canal through the cerebrospinal fluid **(C–F)**.

Radiological data are summarized in Table 1. Based on MRI scans, the lesions were hypointense (*n* = 35, 87.5%) or isointense (*n* = 5, 12.5%) on the T1-weighted images (Figures 1–3), and isointense (*n* = 8, 20.0%) (Figures 1, 2) or hyperintense (*n* = 32, 80.0%) on the T2-weighted images. Thirty-five (87.5%) lesions showed significant heterogeneous enhancement (Figures 1–3) and five (12.5%) lesions showed significant homogeneous enhancement on MRI scans. The lesions involved the cervical spine in six (15.0%) cases, thoracic spine in 24 (60.0%) cases, lumber spine in 13 (32.5%) cases, and sacrum in five (12.5%) cases, respectively. Among these cases, one case showed involvement of both the cervical and thoracic spines, three cases showed involvement of both the sacral and lumbar spines, and four cases showed involvement of both the thoracic and lumbar spines. In addition, tumor lesions involved a single segment in seven (17.5%) cases, and multiple segments in 33 (82.5%) cases. Seventeen patients were radiographed for intraspinal tumors and vertebral bone destruction (Figures 1, 2). Regarding the subtypes, the spinal canal type (Figure 1) was detected in 32 (80.0%) cases and vertebral type (Figure 2) was detected in eight (20.0%) cases.

All of the patients underwent at least one surgery. Partial resection, subtotal resection, total piecemeal resection, and total en bloc resection were performed in four (10.0%) cases, 17 (42.5%) cases, 13 (32.5%) cases, and six (15.0%) cases, respectively. Postoperative radiotherapy was performed in 25 cases, with a median dose of 45 Gy (range, 40–55 Gy). Postoperative chemotherapy was performed in 28 cases.

The mean follow-up period was 23.8 (range, 2–93) months. At the last follow-up, local recurrence occurred in 24 (60%) cases, and seven patients underwent a second operation and one patient underwent a third operation. Distant metastasis occurred in 11 (27.5%) cases. The distant metastatic sites was the lung in six cases, rib in one case, sternum in one case, mediastinum in one case, and spinal cord in two cases (Figure 3).

### Pathology

**Figure 4:**
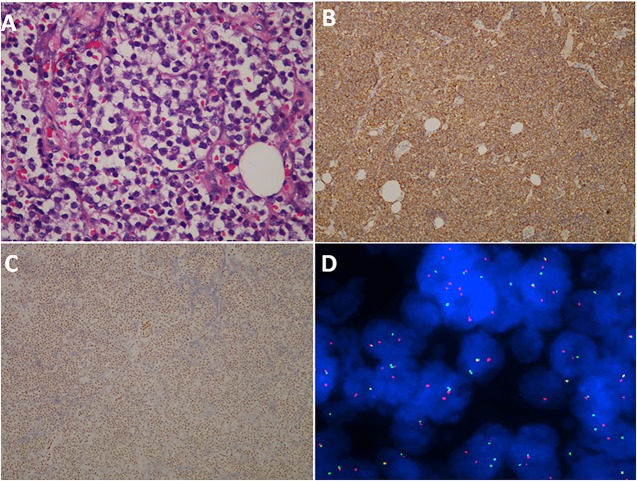
Histopathological, immunohistochemical, and cytogenetic examination of ES/pPNET. Light microscopy showed a highly cellular ES/pPNET tumor consisting of undifferentiated, small, round cells with frequent mitoses **(A)** (hematoxylin–eosin × 400). Immunohistochemical staining showed positivity for CD99 (×100) **(B)**. Microphotograph showing immunohistochemical staining of FLI-1 **(C)**. The representative FISH result using EWSR1 (22q12) dual color break apart rearrangement probe (Vysis). Tumor cells of the ES/pPNET displayed one fusion (yellow signal), and the simultaneous split pattern of one orange and one green signal, being indicative of a rearrangement of one copy of the EWSR1 gene **(D)**.

Light microscopy revealed that the tumor nodule was mainly composed of small, round, undifferentiated cells with hyperchromatic nuclei and reduced cytoplasmic volume (Figure 4). Immunohistochemical studies showed that 40 cases were positive for CD99 (Figure 4). Vimentin was positive in 25 (62.5%) cases. Strong immunoreactivity for Friend Leukemia Virus Integration 1 (FLI-1) was detected in 27 (67.5%) patients (Figure 4). The average Ki-67 labeling index was 30% (range, 3–80%). Furthermore, a fluorescent *in situ* hybridization (FISH) study was performed in two cases, and EWS/FLI1 translocation was found to be present (Figure 4). However, a corresponding FISH study was not performed in the other 38 cases.

### Univariate and Multivariate Analysis of Prognostic Factors for Progression-Free Survival

The median PFS was 14 months. The 1-, 2-, and 5-year PFS rates were 57.7, 30.4, and 9.5%, respectively. The univariate analysis of prognostic factors affecting PFS is presented in Table 2. In the present study, we applied the four following surgical treatment: partial resection, subtotal resection, total piecemeal resection, and total en bloc resection. The PFS rate was statistically significant difference among the four kinds of resection modes (*p* < 0.001). The PFS rate was significantly higher in patients with adjuvant radiotherapy than that of patients without adjuvant radiotherapy (*p* < 0.001). Patients who underwent chemotherapy had a significantly higher PFS rate than those of patients treated without chemotherapy (*p* = 0.016). In addition, the PFS rate was significantly lower in patients with postoperative Frankel score (A–C) than that of those with postoperative Frankel score (D–E) (*p* = 0.019). There were no significant differences among the other factors (i.e., age, gender, disease duration, preoperative Frankel score, subtype, involved segments, Ki-67 index, intraoperative blood loss, and bone destruction).

**Figure 5:**
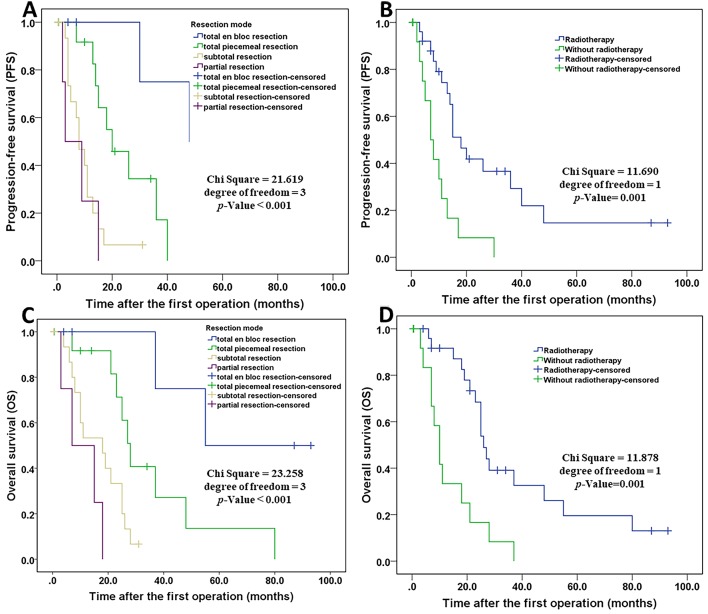
Kaplan–Meier curves of progression-free survival and overall survival. Kaplan–Meier curves of progression-free survival for resection mode **(A)**. Kaplan–Meier curves of progression-free survival for patients treated with radiotherapy and without radiotherapy **(B)**. Kaplan–Meier curves of overall survival for resection mode **(C)**. Kaplan–Meier curves of overall survival for patients treated with radiotherapy and without radiotherapy **(D)**.

Possible prognostic factors, extracted by the univariate analysis, were subjected to the multivariate analysis (Table 3). Multivariate analysis showed that resection mode (*p* < 0.001) and adjuvant radiotherapy (*p* < 0.001) were independent prognostic indicators. The Kaplan-Meier curve of PFS for resection mode and adjuvant radiotherapy are shown in Figure 5. Multivariate analysis revealed that postoperative Frankel score and adjuvant chemotherapy were not independent prognostic factors for PFS. Detailed results are presented in Table 3.

**Table 3:** Multivariate analysis of prognostic factors for progression-free survival and overall survival.

**Factors**	**PFS**			**OS**		
	**HR**	**95% CI**	***p*-Value**	**HR**	**95% CI**	***p*-Value**
Resection mode	1.083	1.255–10.495	<0.001	0.813	1.243–6.115	<0.001
Adjuvant radiotherapy	0.500	1.583–4.217	0.004	0.454	2.082–5.064	<0.001
Adjuvant chemotherapy	–	–	0.189	–	–	0.813
Postoperative Frankel score	–	–	0.303	–	–	0.762
Distant metastasis	–	–	–	–	–	0.491

*CI indicates confidence interval; HR, hazard ratio*.

### Univariate and Multivariate Analysis of Prognostic Factors for Overall Survival

The results of the univariate analysis of the possible prognostic factors affecting OS are presented in Table 2. The median OS was 25 months. The 1-, 2-, and 5-year OS rates were 74.8, 50.7, and 12.2%, respectively. Univariate analysis shown that a significant difference was observed in patients with resection mode (*p* < 0.001), adjuvant radiotherapy (*p* = 0.001), postoperative Frankel score (A–C/D–E) (*P* = 0.013), adjuvant chemotherapy (*p* = 0.029), and distant metastasis (*p* = 0.036). These prognosis related factors extracted by univariate analysis were submitted to Cox regression analysis (Table 3). Resection mode (*p* = <0.001) and adjuvant radiotherapy (*p* < 0.001) were remained highly significant independent prognostic factors for OS. Details of the above five prognostic factors by multivariate analysis are presented in Table 3. Additionally, the Kaplan-Meier curves of OS for resection mode and adjuvant radiotherapy are shown in Figure 5.

### Complications

Erectile dysfunction occurred in one patient. Leakage of cerebrospinal fluid occurred in four patients and was cured within 1 week by lumbar cistern drainage. Three patients were stricken with pneumonia but recovered after being treated with antibiotics for approximately 1 week. No thrombosis, subcutaneous emphysema, secondary spinal malformation, or internal fixation failure were observed after surgery or during the long-term follow-up.

## Discussion

Primary spinal ES/pPNET is an extremely rare family of malignancies that has an aggressive clinical course with high recurrent potential and poor prognosis (5–8). The special anatomical structure of the spine poses a huge challenge for surgical management of ES/pPNET and increases the postoperative recurrence rate. While preventing recurrence, increasing PFS and OS after initial operation is a significant effort that should be pursued and achieved. Due to the low incidence of primary spinal ES/pPNET, the clinical features and prognostic factors remain unclear. In this study, we performed survival analysis to explore independent prognostic factors related to PFS and OS in patients with primary spinal ES/pPNET. The results indicate that total en bloc resection and adjuvant radiotherapy were independent prognostic factors that can significantly improve PFS and OS for patients with primary spinal ES/pPNET.

In the present study, the average age was 21.9 years, which is slightly greater than that in previous reports (5). Similar to other studies (6, 9), our cohort showed clear male predominance in incidence (male:female ratio = 1.5:1). Limb weakness (70%) and pain (50%), as well as incontinence (20%), were the most common initial symptoms, which is largely consistent with previous reports (5, 9). The mean duration of symptoms before the first operation was 42 days, which is longer than that of previous reports (5, 10). The lesions were generally located in the thoracic spine (60.0%), which is consistent with previous reports (11). However, univariate analysis showed that age, gender, and disease duration were not influential factors for prognosis of patients (all *p* > 0.05).

The ES/pPNET tumor nodule is mainly composed of small, round, undifferentiated cells (5). Accurate diagnoses rely on immunohistochemistry and molecular genetic analysis. Some studies showed that membranous expression of CD99 was detected in 97% of cases, and the most sensitive and specific detection method for the diagnosis of primary spinal ES/pPNET was the combination of CD99 and FLI-1 immunohistochemistry (2, 12, 13). In the present study, positive expression of CD99 was found in 40 (100%) cases, consistent with the diagnosis of ES/pPNET. As has been known, the gold standard for diagnosing ES/pPNETs is the identification of the tumor type-specific fusion genes EWSR1/FLI-1 (2, 14–17). However, FISH studies have only been performed in a small portion of the reported cases in the English literature (9). In our series, a FISH study was performed in two cases, and EWS/FLI-1 translocation was found to be present. In addition, our study showed that the average Ki-67 labeling index was 30% with a range of 3–80%. An association between ki-67 index and PFS or OS was not reported in related studies; however, our statistical analysis determined that ki-67 index was not a potential prognostic factor for PFS and OS (all *p* > 0.05).

Surgical treatment is the first-line treatment for primary spinal ES/pPNET, in terms of preserving functionality, removing lesions, relieving symptoms, controlling local recurrence, and promising prolonged survival (16). Since ES/pPNETs have the character of local infiltration, the local recurrence rate will be high if initial surgery is inadequate. Previous studies have demonstrated that gross total resection can result in better prognosis than subtotal resection (5, 10). In our study cohort, resection mode included partial resection, subtotal resection, total piecemeal resection, and total en bloc resection. Our results shown that patients who underwent total en bloc resection had markedly higher PFS rates and OS rates than those treated by total piecemeal resection, subtotal resection, and partial resection. However, en bloc resection of spinal ES/pPNET with wide margins may be difficult because of residual tumor cells on such vital structures as the dura, spinal cord, major blood vessels, or other critical nerves. Allowing for constraints for achieving total en bloc resection to fulfill wide margins, adjuvant radiotherapy, and/or chemotherapy is a critically important consideration in these patients.

Aside from case reports, there is no retrospective analysis focused on surgical management and prognostic factors for patients with ES/pPNET in the spine (vertebral type). The surgical treatments applicable to the vertebral lesion include the simplest subtotal resection, total piecemeal spondylectomy, and the most complex total en bloc spondylectomy (TES) (18–21). In these series of subtypes, surgical resection and reconstruction of the spine were difficult and TES was challenging. The potential role of radiotherapy and/or chemotherapy is still debatable, and no robust direct evidence of impact in survival has been discovered (16). In the present subtype series, total resection, especially TES, combined with radiotherapy with an intensity 40–55 Gy can significantly improve the PFS and OS rates.

Our statistical analysis indicated that total resection, especially total en bloc resection, led to a better prognosis than without total resection (*p* < 0.001). However, some tumors may still relapse and/or progress to metastasis after total piecemeal resection. In our present study, two patients who underwent total piecemeal resection did not show local recurrence, but did show multiple metastases in the spinal canal after 1 year. The reason may be that piecemeal resection is related to a possibility of cancer cell contamination in the field of surgery. Therefore, total resection, especially total en bloc resection when possible, should be strived for in patients with primary spinal ES/pPNETs to avoid tumor cells contaminating the surgical field and increase PFS and OS.

To our knowledge, our present study is a relatively larger series to date on spinal ES/pPNETs, with the longest follow-up until now; additionally, it is the first such study to focus on prognostic factors for PFS and OS. Nevertheless, there are some limitations. First, this is a retrospective design and, thus, potential biases exist. Second, we only focused on surgical cases, and neglected cases from patients who did not undergo surgery. Third, some patients had a relatively short follow-up, which makes OS appear higher than it may be in actuality.

## Conclusions

Primary spinal ES/pPNETs is a challenging and rare clinical entity given its high local recurrence rate and distant metastasis. Resection mode and adjuvant radiotherapy are independent prognostic factors for primary spinal ES/pPNETs. Total en bloc resection can significantly improve PFS for primary spinal ES/pPNETs and adjuvant radiotherapy is a favorable factor for PFS. Total en bloc resection and adjuvant radiotherapy considerably improve OS for patients with primary spinal ES/pPNETs.

## Data Availability

All datasets generated for this study are included in the manuscript and/or the supplementary files.

## Ethics Statement

Because this study was a retrospective study and did not involve any experimental interventions, according to the rules of the ethics committee of Tongji Hospital, it did not require special ethics approval.

## Author Contributions

YW and JC: study design. JC, ML, YZ, LZ, and FF: data collections. JC and ML: data analysis. JC: writing. All authors reviewed the manuscript.

### Conflict of Interest Statement

The authors declare that the research was conducted in the absence of any commercial or financial relationships that could be construed as a potential conflict of interest.
